# Inhibition of CDK4/6 as Therapeutic Approach for Ovarian Cancer Patients: Current Evidences and Future Perspectives

**DOI:** 10.3390/cancers13123035

**Published:** 2021-06-17

**Authors:** Alessandra Dall’Acqua, Michele Bartoletti, Nastaran Masoudi-Khoram, Roberto Sorio, Fabio Puglisi, Barbara Belletti, Gustavo Baldassarre

**Affiliations:** 1 Molecular Oncology Unit, Centro di Riferimento Oncologico di Aviano (CRO), IRCCS, National Cancer Institute, 33081 Aviano, Italy; alessandra.dallacqua@gmail.com (A.D.); nastaran.masoudi@gmail.com (N.M.-K.); bbelletti@cro.it (B.B.); 2 Medical Oncology and Cancer Prevention Molecular Oncology Unit, Centro di Riferimento Oncologico di Aviano (CRO), IRCCS, National Cancer Institute, 33081 Aviano, Italy; michele.bartoletti@cro.it (M.B.); rsorio@cro.it (R.S.); fabio.puglisi@cro.it (F.P.); 3 Department of Medicine (DAME), University of Udine, 33100 Udine, Italy

**Keywords:** ovarian cancer, CDK4/6 inhibitors, cell cycle progression, combination therapy, DNA damage response

## Abstract

**Simple Summary:**

Altered regulation of the cell cycle is a hallmark of cancer. The recent clinical success of the inhibitors of CDK4 and CDK6 has convincingly demonstrated that targeting cell cycle components may represent an effective anti-cancer strategy, at least in some cancer types. However, possible applications of CDK4/6 inhibitors in patients with ovarian cancer is still under evaluation. Here, we describe the possible biological role of CDK4 and CDK6 complexes in ovarian cancer and provide the rationale for the use of CDK4/6 inhibitors in this pathology, alone or in combination with other drugs. This review, coupling basic, preclinical and clinical research studies, could be of great translational value for investigators attempting to design new clinical trials for the better management of ovarian cancer patients.

**Abstract:**

Alterations in components of the cell-cycle machinery are present in essentially all tumor types. In particular, molecular alterations resulting in dysregulation of the G1 to S phase transition have been observed in almost all human tumors, including ovarian cancer. These alterations have been identified as potential therapeutic targets in several cancer types, thereby stimulating the development of small molecule inhibitors of the cyclin dependent kinases. Among these, CDK4 and CDK6 inhibitors confirmed in clinical trials that CDKs might indeed represent valid therapeutic targets in, at least some, types of cancer. CDK4 and CDK6 inhibitors are now used in clinic for the treatment of patients with estrogen receptor positive metastatic breast cancer and their clinical use is being tested in many other cancer types, alone or in combination with other agents. Here, we review the role of CDK4 and CDK6 complexes in ovarian cancer and propose the possible use of their inhibitors in the treatment of ovarian cancer patients with different types and stages of disease.

## 1. Introduction

In 2000, Hanahan and Weinberg, by putting together the principal advancements of cancer research during the previous 25 years, provided the scientific community with the knowledge of what, at that time, were the hallmarks of cancer [1]. An update of these hallmarks was provided by the same authors in 2011 [2]. The sustained proliferative signaling and the evasion from growth suppression were and remained two of the most typical traits reported in virtually all cancer cells. Both these features are tightly related to an altered regulation of the cell cycle progression. Ten years later, we know that a dysregulated cell cycle is not only a hallmark of cancer, but also one of its main vulnerabilities, targetable by specific biological therapies.

Here, we will focus on the possible roles that the inhibition of two central regulators of cell cycle progression, namely CDK4 and CDK6, may play in the context of ovarian cancer. We will highlight how they have been used so far for the treatment of this disease and what are the future steps that need to be made to possibly expand their success.

## 2. The Role of CDK4 and CDK6 in the Control of Cell Cycle Progression

Cell cycle progression is a tightly controlled process that ensures the correct division of one cell into two daughter cells with the same genetic material [3]. To this aim, a series of enzymatic chain reactions ensures that, after a mitogenic stimuli, a cell may first enter the cell cycle from a status of quiescence, faithfully replicate the DNA, and, finally, segregate the DNA content in the two daughter cells [3].

Three principal families of proteins govern the progression through the mitotic cell cycle: cyclins, cyclin dependent kinases (CDK) and CDK inhibitors (CDKI). The consecutive activation of different CDKs allows the cell exit from a state of quiescence, termed G0, and the entering in the cell cycle in a phase known as G1 (or Gap 1) during which the cell prepares to duplicate its genetic content. This happens during the S (synthesis) phase of the cell cycle that follows the G1 phase and precedes the phase G2 (or Gap 2), in which the cell prepares to divide during mitosis (M phase). Each transition from one phase to the other is regulated by the activation of specific cyclins-CDKs complexes. The complexes containing the D type of cyclins (i.e., D1, D2 and D3) and the CDK 4 and 6 are the first complexes activated by the cell following a mitogenic signal. Their activation is necessary for the cells to exit from the state of quiescence, proceed along the G1 phase and bypass the so-called restriction point, a checkpoint that, once bypassed, commits the cell to complete the mitotic cycle and divide. It is therefore not surprising that all cell cycle proteins involved in the regulation of G1 phase progression are often deregulated in cancer and have been for long time conceived as possible therapeutic targets.

In a state of quiescence or of cell cycle resting (phase G0 or early G1), the CDK4/6 are inactive, bound to the CDKI of the INK4 family (i.e., p15^INK4B^, p16^INK4A^, p18^INK4C^ and p19^INK4D^), and the expression of cyclin Ds very low or absent. Contextually, CDK2, the other CDK involved in the G1 to S phase transition, is maintained in an inactive state bound to the CDKI, p27^KIP1^. Under these conditions, the tumor suppressor Rb (Retinoblastoma protein) is active and binds and represses the transcription factors of the E2F family that, in turn, are necessary to transcribe pro-proliferative genes. Upon mitogenic stimuli (e.g., growth factor stimulation of tyrosine kinase receptors, estrogen receptor activation, etc.), cyclins Ds are rapidly transcribed and p27^KIP1^ is tyrosine phosphorylated by nonreceptor tyrosine kinases, such as Src family members [4,5]. These events allow the formation of an active CDK4/6-cyclin D-p27^KIP1^ complex that translocates in the nucleus and phosphorylates Rb, causing the de-repression of E2F family members. As a consequence, E2F factors transcribe the genes necessary to form an active cyclin E/CDK2 complex that allows bypassing the restriction point and, eventually, commits the cell to complete the division.

It is therefore clear that even a small dysregulation of the INK4, CDK4/6, cyclins D and p27^KIP1^ proteins may profoundly impact on the physiological control of the cell cycle. Accordingly, a great body of evidence has demonstrated that one or more of these genes are altered in most human cancers and their alteration often predicts poorer patients’ prognosis.

A clear input toward the comprehension of the role of these proteins in human cancers comes from the generation and characterization of genetically modified mice, knock out (KO) for one or more of these genes. The description of these models is out of the scope of this review but, as a whole, the picture depicted by these studies has defined some pillars and highlighted several important issues. First, the role of cyclin D1, D2 and D3 seems to be organ specific, with cyclin D1 mostly involved in the development of the mammary gland and the retina [6], cyclin D2 in the development of the gonads [7] and cyclin D3 in the maturation of T lymphocytes [8]. Mice deficient for Cdk4 are viable and only display proliferative defects in specific endocrine cell types and the same is true for Cdk6 KO mice, which develop normally with only minor hematopoietic defects [9]. Interestingly, mice lacking both Cdk4 and Cdk6 die at the end of gestation, due to severe anemia, although these embryos display normal organogenesis and most cell types seem to proliferate normally [9]. Very similar results were obtained by the analysis of mice null for the three cyclin Ds [10]. These results indicate that CDK4 and CDK6 have many overlapping functions and that they are not essential for cell cycle entry, since most normal cells are able to activate alternative mechanisms to initiate cell proliferation. Further, these studies suggest that inhibition of CDK4 and CDK6 could be exploited to specifically control the proliferation of tumor cells that rely on their activity.

This hypothesis has been experimentally validated in mice, demonstrating, for instance, that mice lacking cyclin D1, or Cdk4, or mice expressing kinase-deficient cyclin D1-Cdk complexes were resistant to HER2- or Ras-driven mammary tumorigenesis, but not to the one sustained by c-Myc overexpression [11,12]. Accordingly, pharmacological inhibition of Cdk4 and Cdk6 prevented tumor development in mouse models of breast cancer, sustained by cyclin D1, and leukemia, sustained by cyclin D3 [13].

These seminal studies have open the way to the testing of specific CDK4 and CDK6 small molecule inhibitors (hereafter CDK4/6i) to treat human tumors, in particular estrogen receptor positive (ER+) breast cancers, in which their use, combined with hormonal therapies, has proved highly effective in the control of disease progression [14,15,16,17].

## 3. Non-Cell Cycle Dependent Activities of CDK4 and CDK6

Beyond their common activity in governing G1-S cell cycle progression, CDK4 and CDK6 also display kinase dependent and independent functions, therefore regulating many other cellular processes like DNA damage and repair, transcription, senescence, invasion, metabolism and immune response. The discovery of these roles of CDK4 and CDK6 in several cellular processes has certainly positively contributed to the further clinical development of CDK4/6i in different types of cancer, used alone or in combination with other therapeutic approaches [14,18,19,20,21,22,23]. In particular, accumulating evidences indicate that CDK4 and CDK6 play a central and originally unanticipated role in the regulation of the DNA Damage Response (DDR). For instance, it has been shown that CDK4/6 inhibition combined with ionizing radiation results in a shift from homologous recombination (HR) to error-prone non-homologous end-joining (NHEJ) DNA repair mechanism in pancreatic adenocarcinoma, ER+ BC and TNBC models [24,25,26]. Similarly, CDK6 expression protects epithelial ovarian cancer cells from platinum-induced cell death controlling ATR transcription through FOXO3a phosphorylation and stabilization, directly impacting on DDR during the S phase of the cell cycle [27].

More recently, transcriptome analyses performed in different cancer cell models modified for CDK4 and CDK6 expression or activity, highlighted that, beyond the control of the G1 phase of the cell cycle, CDK4 and CDK6 might play non-cell cycle related functions via the regulation of transcription [28]. In particular, it was reported that while CDK4 controls pro-metastatic inflammatory pathways, CDK6 mainly regulates DNA damage, repair and replication, through the modulation of genes, such as TK1, POLD3, POLE2, CENPI and DTL, essential for these processes [28]. Similarly, Watt and colleagues observed that breast cancer cell lines and PDX models treated with the CDK4/6i abemaciclib or palbociclib, displayed a remodeling of the chromatin architecture with a wide activation of transcription enhancers and super-enhancers. These transcriptional modifications are involved in the regulation of luminal differentiation, apoptosis and immune response [29]. Whether these activities of CDK4/6i are due to unexpected off target effects of the inhibitors or effectively to the inhibition of CDK4 and/or CDK6 kinases activities, although largely expected, has not been formally demonstrated [29].

Regulation of gene transcription by CDK4 and CDK6 has a well-established role in the regulation of senescence, through the phosphorylation of the transcription factor FOXM1 and described as an important event in melanoma and ovarian cancer [30,31]. FOXM1 has been identified as a CDK4 and CDK6 substrate using an unbiased phospho-proteomic screening. This approach also highlighted that CDK4- and CDK6-specific target proteins exist [30]. Among others, FOXO3a and several splicing factors seems to be CDK6-specific substrates, supporting the hypothesis that this kinase might regulate not only gene transcription, but also DDR and RNA splicing [27,30].

CDK4 and CDK6 were also associated with the regulation of protein ubiquitination and stability. In particular, it has been demonstrated that both these CDKs can bind and phosphorylate the deubiquitinase USP51 and DUB3, to control ZEB1 [32] and SNAIL1 [33] expression, respectively, thereby modulating the metastatic phenotype of cancer cells. Interestingly, ZEB1 is also a direct phosphorylation target of CDK6 [27,30], suggesting that multiple layers of control exist between CDK4/6 activity and the regulation of cell invasion. Accordingly, it has been shown that the CDK4-cyclin D1 complex could control cell adhesion to the Extra-Cellular Matrix (ECM) and cell motility by directly phosphorylating the Paxillin-Rac1 complex in the membrane ruffles [34].

Intriguing evidences also suggest that CDK4/6 activity is implicated in cell metabolism, as demonstrated by the increase in oxidative phosphorylation when the Rb pathway is inhibited in pancreatic cancers [35]. At mechanistic level, CDK4/6i lead to the compensatory activation of the MEK and mTOR pathways. Accordingly, combined pharmacological inhibition of CDK4/6 and MEK potentiated the cytostatic effect of CDK4/6i, while the use of CDK4/6i *plus* mTOR pathway inhibitors increased cell death [35]. These observations might have high translational relevance, since both MEK and mTOR pathway inhibitors are currently used in clinic for the treatment of several types of human cancers.

Last but not least, evidences accumulated in the last few years highlighted a role for CDK4 and CDK6 inhibition in triggering anti-tumor immunity. The first evidences were obtained in breast cancer with abemaciclib, suggesting that suppression of the Rb–E2F axis leads to a reduced expression of the methyl-transferase DNMT1 and, thus, to the hypomethylation of genes that regulate the immune response. As a consequence, CDK4/6i increased antigen presentation by tumor cells and reduced tumor infiltration by immunosuppressive regulatory T cells [36]. These observations support the possibility that the combined use of CDK4/6i and immune checkpoint inhibitors (e.g., anti-PD-L1 antibodies) may result in synergistic antitumor activity [36,37]. Interestingly, it has been also demonstrated that the cyclin D/CDK4 complex might regulate PD-L1 protein abundance, leading to its proteasomal degradation and that CDK4/6i increases PD-L1 levels in vivo, in breast cancer models [38]. Relevant to this review, these observations were recently confirmed in ovarian cancer models, in which treatment with abemaciclib increases both immune infiltration and the activity of cytotoxic lymphocytes, making ovarian cancer more sensitive to the PD-1 blockade [39].

## 4. Mechanism of Action of CDK4/6 Inhibitors

Targeting the CDK activity as an anticancer strategy has been tested since the late 90s, when the toxicity profile of a pan-CDK inhibitor (flavopiridol) was tested in 76 patients with refractory malignancies [40]. Yet, the way of administration (i.e., intravenous injection), the low therapeutic index and the high toxicity profiles, at the concentrations necessary to inhibit their targets, as already observed for other pan-CDK inhibitors, such as roscovitine, cooled the enthusiasm for this type of targeted therapies [41]. Similarly, the second generation of more selective and more potent CDK inhibitors, such as dinaciclib, demonstrated little clinical activity in several cancer types [41].

In 2001, the first of a kind CDK4/6 specific inhibitor (i.e., PD0332991, then renamed palbociclib) was developed as an orally available pyrido [2,3-d]pyrimidines derivate and proved the ability to block cancer cells in the G1 phase of the cell cycle [42,43]. This small molecule inhibitor is an ATP-competitive inhibitor of CDK4/6-cyclin D, inhibiting these complexes with exquisite selectivity, 20-fold higher than CDK2/cyclin E complex and more than 100-fold higher than FGFR [42,43].

Then, experiments in cells and mouse models of cancer confirmed that PD0332991 displayed very promising antitumor activities [13,43,44], but it took many years until its therapeutic value became really appreciated, as described by Garber in 2014 [45].

The unexpected and impressive activity reported for palbociclib in breast cancer patients when used in combination with anti-estrogen therapy [45] then rapidly stimulated the design of other CDK4/6 inhibitors that rapidly entered in clinical development. Three orally available CDK4/6 inhibitors are currently approved for the treatment of patients with metastatic breast cancer (i.e., palbociclib, ribociclib and abemaciclib) [41] and one, administered intravenously and with shorter half-life, has been developed to prevent chemotherapy-induced myelosuppression [46]. Many others are under clinical development and have been recently reviewed elsewhere [47]. Here, we will focus on the activity of the orally available compounds that have been shown to bind both the monomeric CDK4/6 kinase or the CDK4/6-cyclin D complexes and, by binding the CDK4/6 in the cleft between the N-terminal and C-terminal lobes, inhibit the ATP binding [48]. An important distinctive feature is that abemaciclib, compared to palbociclib and ribociclib, displays a wider spectrum of action and inhibits at nanomolar concentration not only CDK4 and CDK6, but also CDK9 (Table 1).

According to the pivotal role of CDK4 and CDK6 in driving cell cycle progression through the G1 phase of the cell cycle, the most prominent effect of all CDK4/6i is the block of cell proliferation [41]. Biochemically, this cell cycle blockage is accompanied by the inhibition of Rb phosphorylation, an event that can be observed both in vitro and in vivo [41,49]. Therefore, it has been postulated that tumors with alteration in proteins regulating the progression through the G1 phase of the cell cycle will be the most sensitive to these inhibitors. Deletion of Rb family genes and/or amplification of CDK4, CDK6 or cyclin Ds have been proposed as relevant markers of resistance to these drugs [41,47]. However, in line with the multiple activities of CDK4 and CDK6, it is highly expected that the use and the success of CDK4/6i will be also related to many other alterations, present both in tumor cells and in tumor microenvironment.

## 5. Expression of CDK4 and CDK6 Containing Complexes in Ovarian Cancer

Cell cycle proteins are deregulated in almost all types of human cancers, including epithelial ovarian carcinomas [41]. Interestingly, it has been observed that in High Grade Serous Ovarian Cancer (HGSOC) amplification of cyclin E1 (CCNE1) is associated with resistance to platinum-based chemotherapies [50], suggesting that targeting the cell cycle in these patients could represent an effective therapeutic approach.

Here, we have reviewed the studies reporting the expression of the principal regulators of the G1 to S phase transition of the cell cycle. Considering these data as whole, we found that a significant fraction of ovarian cancers that have been analyzed display an aberrant expression of cyclins, CDKs and/or CDKI (Table 2), supporting the hypothesis that these tumors could be potentially sensitive to CDK4/6i.

Slamon and colleagues firstly tried to identify which ovarian cancer type may be sensitive to the CDK4/6i [85]. For this aim, they screened 40 ovarian cancer cell lines for their sensitivity to palbociclib and identified the so-called Rb1-proficient cell lines, with low p16^INK4A^ and CCNE1 expression, as the most responsive to CDK4/6i. They next analyzed the expression of other regulators of CDK4 and CDK6 signaling, including the CDKIs p15^INK4B^, p18^INK4C^, p19^INK4D^, p21^WAF1^ and p27^KIP1^, the Rb family of proteins, Rb2/p130 and Rb3/p107, the D-cyclins (D1, D2 and D3) and the E2F1 transcription factor. However, none of the above proteins strongly correlated with in vitro sensitivity to palbociclib. Conversely, they found an association between cyclin D1 gene (CCND1) amplification and resistance to palbociclib [85]. The analysis of the expression of Rb1 and p16^INK4A^ in a panel of 263 epithelial ovarian cancer samples, by immunohistochemistry (IHC), demonstrated that low/null expression of p16^INK4A^ and Rb1 were associated with shorter patients’ progression free survival [85] and suggested that these tumors could be the ones most benefitting from the treatment with CDK4/6i, alone or in combination with chemotherapy.

We and others have evaluated the expression of both CDK4 and CDK6 in epithelial ovarian cancers with interesting results. In general, it was observed that CDK6, more than CDK4, was expressed at high levels in epithelial ovarian cancer [27,55,76,77,78]. Many evidences suggest that CDK6 regulates the sensitivity to platinum in ovarian cancer cells and that its high expression is associated with a platinum-resistant phenotype [27,77]. Interestingly, CDK6 was mostly localized in the cytoplasm although this localization does not correlate with patients’ survival [78]. In line with this finding, data from publicly available gene expression profile (GEP) datasets suggest that high CDK6, but not CDK4, mRNA expression predicts shorter progression free survival in ovarian cancer patients [27]. Similar observation has been made for D type cyclins, with a clear difference between cyclin D1 (no prognostic role) and cyclin D3, whose high expression predicts shorter patients’ overall/progression free survival (OS/PFS) [27]. However, the results on mRNA expression were not always confirmed by studies that looked at protein expression (Table 2), suggesting that the significance of the expression of D type cyclins in ovarian cancer should be better evaluated. It is interesting to note that cyclin D2, which is a FSH responding gene necessary for gonadal cell proliferation [7], seems to be more and/or exclusively expressed in ovarian germ cell tumors [59,60].

Almost all reported studies suggest that CCNE1 amplification and overexpression predicts shorter patients’ survival (Table 2), in line with the original observation that it is associated with resistance to platinum-based therapy in HGSOC [50].

From studies that analyzed CDKI expression in ovarian cancer samples, it appears clear that the CDKN1A gene (p16^INKA^) had the most relevant prognostic significance. More than one study points to p16^INK4A^ expression as a biomarker of longer patients’ survival (Table 2). Interesting results were obtained for both CDKN1A (p21^WAF1^) and CDKN1B (p27^KIP1^), whose expression was generally associated with lower grade and better patients’ outcome (Table 2). Interestingly, p21^WAF1^ expression seems to be restricted to tumors with wild type TP53 gene [80] and loss of p27^KIP1^ expression has been reported to be an early event in the development of HGSOC [93]. Therefore, it is likely that in HGSOC, in which the first event during transformation is usually the acquisition of a mutation of TP53 gene, both these CDKI are inactivated, eventually leading to higher activity of CDK complexes.

The expression of the others members of the INK4 family of CDKI (CDKN2B, C and D) has been less studied in ovarian cancer (Table 2) but it has been surprisingly observed that CDKN2D (p19^INK4D^) expression was associated with higher tumor stage and shorter patients’ survival [92], which might deserve further investigation in future studies.

## 6. Combination Strategies of CDK4/6 Inhibitors with Conventional Cytotoxic Agents

Most of chemotherapeutics currently used as anticancer agents exert their main effect by targeting actively proliferating cells. In particular, platinum salts, anthracyclines and topoisomerase inhibitors are mostly active during the S phase of the cell cycle, while DNA is duplicated, whereas taxanes, vinca alkaloids and eribulin mostly target the mitotic process during the M phase. Accordingly, the side effects of these conventional cytotoxic agents are particularly evident in healthy cells that are highly proliferating in organs and tissues, such as the bone marrow, the epithelial tissues of the gastrointestinal tract and the skin.

Since CDK4/6i block both normal and cancer cells in the G0/G1 phase of the cell cycle, it was firstly hypothesized that the use of these novel agents in combination with standard chemotherapies could be detrimental for the efficacy of the cancer treatment [94]. Nevertheless, a growing body of preclinical evidence now supports the sequential use of standard chemotherapeutics followed by CDK4/6 inhibitors [95] and, as consequence, early phase clinical trials testing these combinations are thriving. This drastic route change is mainly due to the recent discoveries regarding the role played by CDK4/6 in DDR. These new data in fact suggest that CDK4/6 inhibitors may well cooperate with DNA or mitotic damaging agents to enhance their anti-tumor activity, if used with the proper time schedule [47].

In ovarian cancer, platinum salts are the most commonly used and active agents for patients with newly diagnosed disease, as well as for those with a platinum-sensitive recurrent disease [96]. It is well known that platinum induces the formation of DNA single and double strand breaks, leading to the activation of DDR. It is also recognized that the proper activation of this cellular response, in which the ATR kinase plays a central role, often represents a cause of escape from platinum-induced apoptosis [97,98]. Notably, recent studies from our lab have demonstrated that CDK6, mainly in complex with cyclin D3, contributes to the activation of DDR in HGSOC and induces the expression of ATR [27]. As expectable, the activation of this survival mechanism eventually protects TP53 deficient ovarian cancer cells from platinum-induced cell death. Accordingly, CDK6 inhibition with palbociclib significantly increased platinum-induced apoptosis both in vitro and in vivo [27]. Similarly, it was reported that ribociclib showed synergism with platinum, in xenograft models of HGSOC exposed to concurrent ribociclib and cisplatin treatment, followed by maintenance with ribociclib [99]. On the basis of these promising preclinical results, the use of CDK4/6i to increase the platinum efficacy in ovarian cancer patients is currently being tested in several clinical trials (Table 3).

Based on recent studies and in line with what just mentioned above, it is also possible that inhibition of CDK4/6 may have synergistic activity with inhibition of Poly ADP-ribose polymerase (PARPi). Since PARPi increases genomic instability in cancer cells and CDK4/6i impair the DDR, this strategy could induce HR deficiency, even in HR proficient ovarian cancers. Recent evidences in preclinical ovarian cancer models, combining palbociclib with the PARPi olaparib, have indeed demonstrated a clear synergism in ovarian cancers with high Myc expression [100]. The clinical synergism between PARPi and CDK4/6i is now under clinical investigation in different cancer settings (Table 3).

## 7. Use of CDK4/6 Inhibitors as Single Agents in Ovarian Cancer Patients

The effectiveness of the CDK4/6 inhibitors as single agent (palbociclib) was explored in a phase II trial in heavily pretreated ovarian cancer patients. Efficacy was assessed in 30 patients, mainly serous ovarian cancer patients, showing 9/30 (30%) patients that were progression-free at 6 months. The authors reported one partial response and 17 disease stabilizations, with a median PFS of 3.7 months. Palbociclib was well tolerated, and hematological toxicities of grade 3 and 4 were reported in only 10 patients [101]. Based on the preclinical data collected by the same group, the expression of Rb1, CDKN2A and/or CCNE1, along with the amplification of CCND1, could represent good biomarkers to predict response [85]. These biomarkers are absolutely necessary, since the activity of palbociclib in an unselected population seems to be modest, at least when it is administered as single agent.

The association between CDK4/6i with anti-hormonal therapies certainly deserves to be mentioned for its promising results in gynecological malignancy, particularly in hormone receptor positive endometrial and ovarian cancers where endocrine agents are generally used alone in later treatment lines. A trial combining ribociclib and letrozole in estrogen receptor positive (>10%) endometrial and ovarian cancer patients demonstrated promising clinical activity in relapsed ovarian cancer patients [102]. About 50% of the 40 patients enrolled were progression-free after 12 weeks and, interestingly, the greatest benefit was seen in low grade serous ovarian cancer (LGSOC) patients, who had PFS longer than 24 months. However, since only 3 LGSOC patients were enrolled in the trial, these data will need further confirmation [102].

## 8. Clinical Experiences on the Use of CDK 4/6i with Chemotherapy

The combination of platinum-based chemotherapy with CDK4/6i has been tested in several solid malignancies. The study by Swiecicki et al. was of particular interest because the combination was specifically tested to improve the efficacy of platinum [103]. This phase II trial enrolled recurrent and metastatic head and neck cancer patients, treated with carboplatin on day 1 (with a starting dose of 5AUC) and palbociclib (125 mg daily) on days 1–14, every three weeks. This schedule of carboplatin and palbociclib showed disappointing antitumor activity, with a 12-week disease control rate of 33% (5/18 stable disease and 1/18 partial response), a median PFS of 2.9 months and significant associated toxicities: grade 3 or higher toxicities were seen in 79% of patients, with the most common being myelosuppression [103]. It is conceivable that this weak anti-tumor activity is due, at least in part, to an incorrect timing of palbociclib administration: given in concomitance with platinum and not sequentially, palbociclib may not be able to prevent the recovery from cytotoxic DNA damage. In conclusion, the treatment schedule proposed by the Swiecicki et al. was suboptimal not only for the lack of a synergistic effect between the two drugs, but also for the high rate of toxicity that could have mined dose intensity. Best dose/sequence finding studies are greatly needed to overcome these limitations.

In another study, the CDK4/6i trilaciclib was used in combination with chemotherapy in breast cancer patients with metastatic triple negative disease, in order to reduce dose-limiting hematological and myeloid-toxicities of chemotherapy [104]. Intriguingly, despite the fact that addition of trilaciclib failed to protect immune cells and bone marrow from chemotherapy-induced damage, the arms in which patients received trilaciclib showed a clinically meaningful survival advantage compared to the arm treated with chemotherapy alone (median OS was 12.6 months with chemotherapy alone, 20 months and 17.8 months in the two trilaciclib arms) [104]. Major limitations of the trial were the small sample size and the open label design. Nevertheless, the possibility that this difference in survival could be linked to a synergistic effect of chemotherapies with the CDK4/6i certainly deserves further investigation.

An intermitting schedule of palbociclib followed by paclitaxel was evaluated in a phase 1 study in breast cancer patients [24]. The trial stemmed from promising preclinical results and tested the possibility to enhance treatment efficacy by using intermittent, alternating dosing with palbociclib and paclitaxel, in 27 metastatic Rb1-proficient breast cancer patients [105]. The recommended dose and schedule found was 75 mg of palbociclib administered for 3 consecutive days, started at least 24 h after each dose of weekly paclitaxel. The clinical benefit rate was 55% at the recommended schedule and it was observed across all receptor subtypes. Thanks to these results, the association of palbociclib with paclitaxel is currently under investigation also in a phase 1 trial, in patients with advanced pancreatic cancer (NCT02501902).

## 9. CDK4/6 Inhibitors Safety and Tolerability Profile

Pivotal phase III trials in advanced breast cancer, where CDK4/6 inhibitors were administered in combination with endocrine therapy (e.g., aromatase inhibitor, fulvestrant), have revealed that palbociclib, ribociclib and abemaciclib were generally well tolerated. In these trials, as well as in clinical practice, adverse events were easily manageable with treatment delay, dose modification and supportive care measures.

Regarding class-specific side effects, which are commonly seen with all three inhibitors, hematological toxicities, in particular neutropenia, are most frequent with palbociclib and ribociclib than abemaciclib.

Regarding drug-specific side effects associated with different CDK4/6 inhibitors, ribociclib might cause hepatotoxicity and has also been linked with reversible prolongation of the QT interval. On the other hand, abemaciclib can induce diarrhea more frequently than palbociclib and ribociclib and in the MONARCH studies venous thromboembolic events were found more frequently in the abemaciclib than in the placebo group. Finally, since all three molecules are substrates of the CYP3A4 enzyme, concomitant therapies with moderate or strong inhibitors or inducers of CYP3A4 must be avoided.

## 10. Conclusions and Future Perspectives

CDK4/6i have proven to be highly effective in the treatment of advanced stage estrogen receptor-positive HER2-negative breast cancer and, in these patients, the combination of CDK4/6i and hormonal therapy represents now the standard of care [106].

Based on this evident clinical benefit, the activity of CDK4/6i as single agent or in combination therapies is being tested also in other types of cancer.

**Figure 1:**
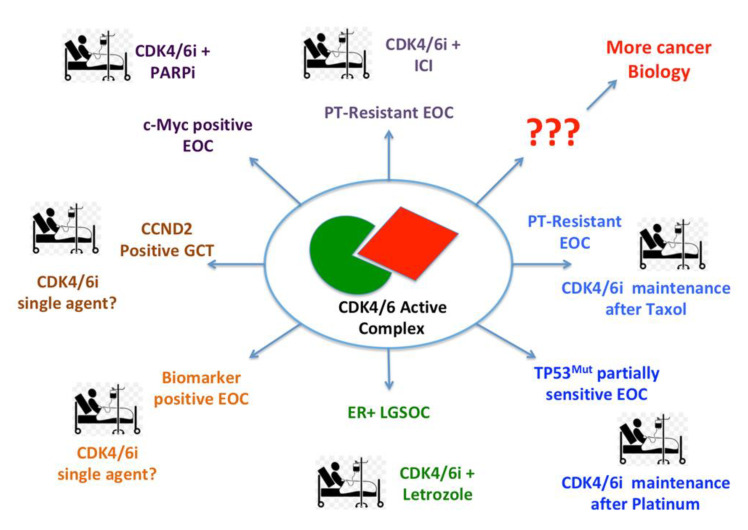
Possible therapeutic approaches based on the use of CDK4/6i in ovarian cancer patients.

Ovarian cancer represents a complex and extremely heterogeneous group of neoplasms. Based on accumulating preclinical and clinical evidences, the use of CDK4/6i could be successfully tested in several settings. At this regard, it is to note that CDK4/6 signaling is frequently altered in these tumors, although, depending on the histotype considered, the specific altered gene may vary (see above). Keeping this concept in mind and if they will be tested appropriately, looking at the different disease grades, stages, histologies and molecular alterations, we expect that the use of CDK4/6i will be of great promise also for ovarian cancer patients (Figure 1).

Accumulating preclinical and clinical evidences suggest that CDK4/6i could be used alone and in association with other chemo- or targeted-therapies in different tumor contexts. Here, we propose possible applications for ovarian cancer patients with different types/stages of disease (see text for detail). EOC = Epithelial Ovarian Cancer; GCT = Germ Cell Tumors; LGSOC = Low Grade Serous Ovarian Cancer; PARPi = PARPs (Poly ADP Ribose Polymerases) inhibitors; ICI = Immune Checkpoint Inhibitors; PT = Platinum; ER+ = Estrogen Receptor positive.

The use of CDK4/6i as single agents in ovarian cancer patients does not seem to hold promise and is probably not worth pursuing further, unless specific biomarkers of activity will be identified. At this regard, based on the biology of germ cell tumors (GCT) and on the fact that they usually overexpress cyclin D2, it could be worth testing if CDK4/6i might be particularly active in these histotypes. Indeed palbociclib seems to be active in a subset of patients with GCT [107] and novel in vitro experiments support a dual role for CDK4/6i in controlling GCT cell proliferation and survival [108]. However, since these rare germinal tumors usually affect adolescents or young adults, the set-up of a clinical trial will be particularly complicated. On the other hand, for the same reason, it would be particularly relevant sparing whenever possible the adverse effects of chemotherapy to these young patients.

The results of the phase II trial, testing the efficacy of ribociclib and letrozole in patients with recurrent ovarian cancer and showing a high response rate in the few patients with low grade tumors, absolutely merit to be further explored, broadening the cohort of LGSOC patients.

In HGSOC, which almost invariably carry mutations in the tumor suppressor gene TP53 and, for this reason, are predicted to be highly sensitive to ATR inhibition [27,109], it would be worth testing the association between CDK4/6i and cisplatin, using CDKi as maintenance therapy. This schedule should, in principle, allow the strongest synergistic effect and avoid the accumulation of hematological toxicities, as observed in head and neck patients that were concomitantly treated with carboplatin and palbociclib [103]. This regimen could be promising also in recurrent ovarian cancer patients that have been treated with platinum-based therapy and still display partial response to platinum. In platinum resistant disease, based on the recent evidences obtained in pancreatic cancer models [25], it would be worth testing the sequential administration of taxanes followed by CDK4/6i as maintenance therapy.

In the setting of ovarian cancer patients with c-Myc overexpressing tumors it would be important testing if the association of CDK4/6i with PARPi is really effective, as demonstrated in preclinical models [100]. This regimen would have the advantage of being a chemo-free approach, particularly indicated for frail patients.

No mature evidence currently supports the adoption of immunotherapy in ovarian cancer patients [110]. Since CDK4/6i can profoundly impact on the immune response to different cancer types, it would be interesting to verify if the use of CDK4/6i may improve the efficacy of immune-checkpoint inhibitors, which would be particularly needed for platinum resistant patients that still have very few valid therapeutic options.

From all the studies conducted so far, we can undoubtedly conclude that the design and management of these novel clinical trials will only be possible in the frame of large, cooperative, translational, multidisciplinary groups, dedicated to the research and the care of gynecological tumors, of which ovarian cancers are not the most frequent but certainly still the most deadly ones.

## Figures and Tables

**Table 1:** Activity of orally available CDK4/6 inhibitors on cyclin/CDKs complexes, in vitro.

Cyclin/CDK	Palbociclib	Ribociclib	Abemaciclib
cycD1/CDK4	11 nM	10 nM	2 nM
cycD3/CDK4	9 nM		
CDK6	15 nM	39 nM	9.9 nM
cycA-E/CDK2	>20 mM	>50 mM	0.5 mM
cycT1/CDK9	NR	NR	57 nM
p25/CDK5	>40 mM	>10 mM	0.3 mM

Information have been retrieved from [41,42,43,49]: Abbreviations: NR, Not Reported, nM, nanoMolar.

**Table 2:** Expression of cell cycle proteins in ovarian cancer.

Gene (Protein)	Histotype	Number of Cases	Technique Used	Notes	Ref
*CCND1* (cyclin D1)	EC, CCC, MC, SOC, Poorly differentiated	43	IHC	Overexpression in 26% of borderline and low-grade tumor samples	[51]
	EC, CCC, MC, SOC, Mixed	81	IHC, Western blot, RFLP-PCR	Amplification and overexpression not related to tumor stage or patients’ survival	[52]
	Benign, Borderline, EC, CCC, MC, SOC, Undifferentiated	79	IHC	High-level of Cyclin D1 in borderline and low grade tumors	[53]
	Serous EOC	134	IHC	Increased expression predicts shorter OS. Inverse correlation between CCND1 and CDKN1B expression	[54]
	EC, MC, SOC, Undifferentiated, other	65	Southern and, Northern blot	Increased expression in 18% of cases; no impact on PSF	[55]
	Advanced serous EOC	66	IHC	High expression predicts shorter patients’ PSF and OS	[56]
	LGSOC (*n* = 26) HGSOC (*n* = 34)	60	IHC	Expressed in 67% of HGSOC samples. Expression predicts shorter OS	[57]
	LGSOC (*n* = 27) HGSOC (*n* = 23)	50	IHC	Upregulation observed in HGOSC and FIGO stage III; high expression predicts shorter patients’ OS	[58]
	EOC	1307	GEP	mRNA expression not related to patients’ survival	[27]
*CCND2* (cyclin D2)	EC, GCT, SOC, Normal OV	24	RT-PCR	mRNA overexpressed in GCT	[59]
	GCT	78	RT-PCR IHC	Protein overexpressed in 42% of analyzed samples	[60]
	EC, CCC, MC, SOC	71	MS-PCR	Promoter hyper-methylation associated with advance stage, residual tumor size, and shorter PSF	[61]
	Well, moderate poor differentiated	92	RT-PCR	Higher expression in tumors respect to normal tissues	[62]
*CCND3* (cyclin D3)	EC, CC, MC, SOC, poorly differentiated	109	IHC	Expression decreased in high grade/high stage tumors; absent expression predicts poor survival	[63]
	EOC	1307	GEP	High mRNA expression predicts shorter patients’ survival	[27]
*CCNE1* (cyclin E1)	Benign, Borderline, SOC, MC, EC, CCC Undifferentiated	103	IHC, Western blot	High expression predicts shorter patients’ OS	[64]
	Serous EOC	134	IHC	Overexpression increased with tumor grade	[54]
	SOC, MC, EC, CCC	88	IHC, FISH	Amplification associated with higher tumor grade and stage predicts shorter patients’ PSF and OS	[65]
	Serous EOC	172	RT-PCR	High expression predicts shorter patients’ OS	[66]
	Normal, Benign, SOC, MC, EC, CCC	117	IHC	Overexpression in 40% of analyzed tumors	[67]
	HGSOC	140	FISH IHC	High expression predicts shorter patients’ OS	[68]
	HGSOC STIC	80	IHC, FISH	Amplification was higher in HGSOC than STIC	[69]
	CCC, EC, SOC	207	IHC, FISH	Amplification and overexpression associated with worse outcome in stage I tumor	[70]
	HGSOC	262	ISH, IHC	Amplification and higher expression predict shorter patients’ OS	[71]
	HGSOC	40	IHC	No relation between CCNE1 level and response neoadjuvant chemotherapy	[72]
	HGSOC	110	IHC	High expression predicts platinum resistance and shorter patients’ OS	[73]
	HGSOC	48	CISH, IHC, Nanostring digital PCR	Amplification and higher expression predict shorter patients’ OS	[74]
*CCNE2* (cyclin E2)	EOC	172	RT-PCR	Amplification and expression had no significant impact on clinical outcome	[66]
*CDK2*	SOC, MC, EC, Undifferentiated	108	Southern blot RT PCR	Amplification in 6.4% of analyzed samples	[75]
	Benign, Borderline, SOC, MC, CCC, EC, Undifferentiated	103	IHC, Western blot,	High expression correlated with high tumor stage and predicts shorter patients’ OS	[64]
*CDK4*	EC, MC, SOC, Undifferentiated, other	48	Southern and Northern blot	Not amplified in the analyzed tumors	[55]
	Benign, Borderline, SOC, MC, EC, CCC, Undifferentiated	103	IHC, Western blot	Overexpressed in malignant tumors. Overexpression associated with low CDKN2A expression and shorter OS	[76]
	EOC	1307	GEP	mRNA expression not related to patients’ survival	[27]
*CDK6*	EOC	30	IHC, RT-PCR	Upregulated in tumors compared to adjacent normal tissue	[77]
	EOC	1307	mRNA	High mRNA expression predicts shorter patients’ survival	[27]
	HG-EOC	73	IHC, Western blot	Overexpression in 74% of analyzed tumors	[27]
	SOC, MC, EC, CCC, Mixed Undifferentiated	223	IHC	High expression in 80% of analyzed tumors. Prevalent cytoplasmic localization	[78]
*CDKN1A* (p21^WAF1^)	EC, CCC, MC, SOC, Mixed	316	IHC	Low expression predicts shorter OS in older patients	[79]
	EC, CCC, MC, SOC, Mixed Undifferentiated	106	IHC	Higher expression in early stage tumor (FIGO I /II), associated with no tumor recurrence	[80]
	EC, CCC, MC, SOC, NOSa, Others	267	IHC	Higher expression in in p53 WT samples predicts longer patients’ OS	[81]
	EC, CCC, MC, SOC, Anaplastic	129	IHC,	Expression higher in CCC lower in MC; no relation with tumor grade, stage or survival	[82]
*CDKN1B* (p27^KIP1^)	Serous Non-serous	88	IHC RT-PCR	Lower nuclear staining and mRNA level in tumor compared to normal tissue; expression associated with lower stages, good prognosis and better response to chemotherapy	[83]
	Not-specified	200	RT-PCR Western blot	Down-regulation of p27 in tumor compared to normal tissues	[84]
*CDKN2A* (p16^INK4A^)	EC, SOC, MC, CCC, Mixed, Undifferentiated	263	IHC	Low expression predicts shorter patients’ OS	[85]
	HGSOC LGSOC	106	IHC	Increased expression of p16^INK4A^ in high grade ovarian tumors	[86]
	EC, SOC, MC, CCC, Mixed, Undifferentiated	190	IHC	High expression in malignant tumors related to shorter patients’ OS	[87]
	EC, SOC, MC, Transitional cell, Undifferentiated	300	IHC	Low expression predicts shorter patients’ OS	[88]
*CDKN2B* (p15^INK4B^)	Serous EOC	52	MS-PCR RT-PCR	Promoter hyper-methylation and lower mRNA expression in cancer compared to normal	[89]
	EC, SOC, MC, CCC, EC, Brenner, GCT	75	MS-PCR	Promoter hyper-methylation in CCC samples	[90]
*CDKN2C* (p18^INK4C^)	GCT	15	RT-PCR	Expressed in all tumors, without any relation to clinic-pathological factors	[91]
*CDKN2D* (p19^INK4D^)	EC, CCC, SOC, Undifferentiated Mixed	445	IHC RT-PCR	High expression in advanced tumor grade or stage associated with shorter patients’ OS	[92]

**Abbreviations**: **Histotype:** CCC, Clear Cell Carcinoma; EC, Endometrial Carcinoma; EOC, Epithelial Ovarian Cancer; GCT, Germ Cell Tumors; HGSOC, High Grade Serous Ovarian Carcinoma; LGSOC, Low Grade Serous Ovarian Carcinoma; MC, Mucinous Carcinoma; Normal OV, Normal Ovary; NOSa, Not Otherwise Specified adenocarcinoma; SOC, Serous Ovarian Carcinoma. **Techniques**: CISH, Chromogenic In Situ Hybridization; FISH, Fluorescent In Situ Hybridization; GEP, Gene Expression Profile; IHC, ImmunoHistoChemistry; MS-PCR, Methylation-specific-Polymerase Chain Reaction; RFLP-PCR, Restriction Fragment Length Polymorphism-Polymerase Chain Reaction; RT-PCR, Reverse Transcriptase -Polymerase Chain Reaction; **Notes**: FIGO, International Federation of Gynecology and Obstetrics; PSF, Progression Free Survival; OS, Overall Survival; STIC, Serous Tubal Intraepithelial Carcinoma.

**Table 3:** Ongoing clinical trials with CDK4/6 inhibitors in ovarian cancer patients.

Title	Phase	Population	Intervention	Primary Endpoint (s)	Status	NCT
Palbociclib With Cisplatin or Carboplatin in Advanced Solid Tumors	I	Solid neoplasms including ovarian cancer	Cisplatin on day 1 and palbociclib on days 2–22. Treatment repeats every 28 days.	Incidence of adverse events; incidence of DLT; RP2D	R	NCT02897375
PF-07104091 as a Single Agent and in Combination Therapy	I/II	Platinum resistant ovarian cancer, advanced breast cancer; NSCLC, SCLC	PF-07104091 (CDK2 inhibitor) administered orally alone or in combination with palbociclib and letrozole.	Incidence of adverse events; incidence of DLT	R	NCT04553133
Ribociclib with Platinum-based Chemotherapy in Recurrent Platinum Sensitive Ovarian Cancer	I	Recurrent platinum sensitive ovarian cancer	Participants will receive 200, 400, or 600 mg of ribociclib per day in combination with carboplatin + paclitaxel. Subjects will receive 6 cycles of carboplatin + paclitaxel given weekly with ribociclib.	MTD	NR	NCT03056833
Ribociclib and Gemcitabine Hydrochloride in Treating Patients With Advanced or Metastatic Solid Tumors	I	Advanced solid neoplasms	Patients receive gemcitabine hydrochloride on days 1 and 8 and ribociclib on days 8–14. Courses repeat every 21 days.	MTD	NR	NCT03237390
Testing the Addition of Abemaciclib to Olaparib for Women With Recurrent Ovarian Cancer	I	Platinum-resistant ovarian cancer	Patients receive olaparib on days 1–28 and abemaciclib on days 8–28 of cycle 1 and days 1–28 of subsequent cycles. Cycles repeat every 28 days.	RP2D	NYR	NCT04633239
Abemaciclib for the Treatment of Recurrent Ovarian or Endometrial Cancer	II	Hormone receptor positive recurrent ovarian or endometrial cancers	Patients receive abemaciclib on days 1–28. Patients with tumors that are hormone receptor positive also receive and anastrozole or letrozole per standard of care. Cycles repeat every 28 days.	Progression-free survival	NYR	NCT04469764

Data refer to the information available on 31 December 2020, in the www.clinicaltrials.gov web site. Abbreviations: R, recruiting; NYR, not yet recruiting; NR, not recruiting; DLT, dose limiting toxicities; RP2D, recommended phase 2 dose; SCLC small cell lung cancer, NSCLC non-small cell lung cancer; MTD, maximal tolerated dose.

## Abbreviations

CCC: Clear Cell Carcinoma; CISH: Chromogenic In Situ Hybridization; EOC: Epithelial Ovarian Cancer; EC: Endometrioid Carcinoma; FISH: Fluorescent In Situ Hybridization; GEP: Gene Expression Profile; GCT: Granulosa Cell Tumors; HGSOC: High Grade Serous Ovarian Carcinoma; IHC: Immunohistochemistry; LGSOC: Low Grade Serous Ovarian Carcinoma; MS-PCR: Methylation-Specific Polymerase Chain Reaction; MC: Mucinous Carcinoma; NOSa: Not Otherwise Specified adenocarcinoma; PSF: Progression Free Survival; OS: Overall Survival; RPPA: Reverse Phase Protein Array; STIC: Serous Tubal Intraepithelial Carcinoma.
